# High pressure and multiferroics materials: a happy marriage

**DOI:** 10.1107/S2052252514020569

**Published:** 2014-10-31

**Authors:** Edmondo Gilioli, Lars Ehm

**Affiliations:** a IMEM-CNR, Area delle Scienze 37/A, Parma 43124, Italy; b Mineral Physics Institute, Stony Brook University, 255 Earth and Space Science Building, Stony Brook, NY 11794-2100, USA; c Photon Sciences Directorate, Brookhaven National Laboratory, 75 Brookhaven Avenue, Upton, NY 11973-500, USA

**Keywords:** high pressure, multiferroics, materials science

## Abstract

The community of material scientists is strongly committed to the research area of multiferroic materials, both for the understanding of the complex mechanisms supporting the multiferroism and for the fabrication of new compounds, potentially suitable for technological applications. The use of high pressure is a powerful tool in synthesizing new multiferroic, in particular magneto-electric phases, where the pressure stabilization of otherwise unstable perovskite-based structural distortions may lead to promising novel metastable compounds. The *in situ* investigation of the high-pressure behavior of multiferroic materials has provided insight into the complex interplay between magnetic and electronic properties and the coupling to structural instabilities.

## Abstract 2

The effect of high pressure on the synthesis, properties and atomic structure of multiferroic materials with perovskite or perovskite-related structures is reviewed.

## 1. Introduction   

Multiferroism can generally be defined as a phenomena in which two or more of the so-called ‘ferroic’ order parameters (ferroelectricity, ferromagnetism and ferroelasticity) simultaneously coexist in a single phase material.

Among the different interactions, magneto-electric coupling is the most desirable property; it refers to either the induction of magnetization by an electric field or polarization by a magnetic field. The ultimate goal is a single phase multiferroic (MF), with *strong coupling* between magnetic and electronic order parameters and the potential to mutually manipulate them at *high (ambient) temperature*. Aside from the very promising technological applications (Nan *et al.*, 2008 ▶), the fascinating fundamental physics of MF materials certainly deserves further investigation.

Unfortunately, these compounds are extremely rare in nature; the scarcity of magneto-electric MFs can be understood by investigating a number of factors, including symmetry and electronic properties. From the structural point of view, there are only 13 point groups that can give rise to multiferroic behavior. Moreover, ferroelectrics by definition are insulators, while itinerant ferromagnets need conduction electrons. There seems to be an intrinsic contradiction between the conventional mechanism of off-centering in a ferroelectric, related to transition metals with *d* ^0^ electronic configuration and the formation of magnetic order which requires the presence of unpaired electrons.

Interestingly, Landau & Lifshitz (1959 ▶) in a volume of their Course of Theoretical Physics stated ‘*Let us point out two more phenomena, which, in principle, could exist. One is piezomagnetism… the other is a linear coupling between magnetic and electric fields in a media… Both these phenomena could exist for certain classes of magnetocrystalline symmetry, but it seems that till present, they have not been observed in any substance*’.

Yet in 2000, Nicola A. Hill published a paper (Hill, 2000 ▶) entitled: ‘Why are there so few magnetic ferroelectrics?’, clearly addressing the complexity of these materials.

Even to probe the magneto-electric coupling is a complicated issue (Bibes, 2012 ▶); it can be measured *indirectly* by simply recording changes in either the magnetization near a ferroelectric transition temperature or the dielectric constant near a magnetic transition temperature (‘magnetocapacitance’ or ‘magnetodielectric’ response). *Direct measurements* are more challenging, requiring either a magnetic response to an applied electric field [*M*(*E*)] or an electrical response to an applied magnetic field [*P*(*H*)].

All these complexities were too puzzling until recently, when the researchers found out that an additional electronic or *structural driving force* must be present for ferromagnetism and ferroelectricity to occur simultaneously.

Undoubtedly, the recent ‘explosion’ in MF studies is enabled by a combination of theoretical and experimental factors. In particular for the latter, new synthesis techniques such as vacuum-based thin film deposition techniques (for hetero-structures) and *high pressure/high temperature* (HP/HT), allow new materials to be obtained by stabilizing metastable or highly distorted structures, which might support ME coupling. Indeed, the use of high-pressure techniques (both for the synthesis and the characterization) is providing important (and somewhat unexpected) results.

Multiferroism is still a relatively young field of research; although predicted in the late 1950s (Landau & Lifshitz, 1959 ▶) and the term coined in 1994 (Schmid, 1994 ▶), the attention raised exponentially only after 2003, the ‘golden year’ for MFs, concomitant with the discovery of large ferroelectric polarization in epitaxial grown thin films of BiFeO_3_ (Wang *et al.*, 2003 ▶) and the discovery of strong magnetic and electric coupling in orthorhombic TbMnO_3_ (Kimura *et al.*, 2003 ▶) and TbMn_2_O_5_ (Hur *et al.*, 2004 ▶). New results are continuously reported and material scientists (and the high-pressure community) are working hard to contradict Landau and Lifshitz’s statement and to prove Hill’s paper to be outdated. A measure of the activity of the research on MF materials is the large number of publications; at the date of submission of the present papers, about 4050 papers (2440 from 2010) were published with the term ‘multiferroic’ in the title and 4200 (1780 from 2010) with the term ‘magneto-electric’ (source: Google Scholar).

Besides the massive literature available on MFs (Spaldin & Fiebig, 2005 ▶; Fiebig, 2005 ▶; Eerenstein *et al.*, 2006 ▶; Khomskii, 2006 ▶; Rao & Serrao, 2007 ▶, and reference therein), this review paper is limited to discuss the role of HP in the study of magneto-electric MF materials, combining HP/HT synthesis (why HP synthesis is so often needed?) and HP characterization (what kind of information can be derived from HP characterization techniques?).

## 2. HP/HT synthesis of bulk MF materials   

The tremendous rise in research on MF and magneto-electric materials pushed the material scientists to search for new materials and mechanisms leading to magneto-electric coupling and multiferroic behavior. Historically, HP/HT synthesis has proven to be very effective in producing a large number of new phases (Badding *et al.*, 1995 ▶; McMillan, 2002 ▶, 2003 ▶; Brazhkin, 2007 ▶), for example in the fields of super-hard materials (Haines *et al.*, 2001 ▶), superconductors (Bos, Penny *et al.*, 2008 ▶) *etc*. For some of them (for example when high density is required, as in the super-hard materials), the role of applied pressure is intuitively easy to understand, while for other systems, often supporting complex electronic, magnetic or transport properties, one can guess that the stabilization of uncommon metastable phases/structures/coordinations offers a pathway to new properties. Indeed, MF materials belong to the latter category.

A detailed description of the possible mechanisms supporting the multiferroism can be easily taken from the literature (see, for example, references cited in Khomskii, 2009 ▶) and goes beyond the scope of the present paper; they can be summarized in *Lone-Pair MF* (Es., BiFeO_3_; Neaton *et al.*, 2005 ▶), *Geometric Ferroelectric MF* (Es.: hexagonal *R*MnO_3_; Van Aken *et al.*, 2004 ▶), *Charge Ordering Ferroelectrics* (Es.: LuFe_2_O_4_; Ikeda *et al.*, 2005 ▶; or (Pr,Ca)MnO_3_; van den Brink & Khomskii, 2008 ▶) or *Spin Spiral Ferroelectrics* (Es.: TbMnO_3_; Kimura *et al.*, 2003 ▶; or TbMn_2_O_5_; Mostovoy, 2006 ▶).

Already from this general list, one should ask: why does the perovskite-type structure play such an important role in the search for MF properties? Generally speaking, it is well known that it is a very common structure, showing an extraordinary variety of properties. Many different atoms can occupy in particular the cationic sites, allowing a wide tuning of their properties. Moreover, the majority of ferroelectric materials exhibit perovskite structure.

People working with high pressure (and particularly geologists) are familiar with perovskites; being a typical HP structure (dense packing, high coordination number, low inter-atomic distances *etc.*) it is commonly found in our planet starting from the mantle to the core–mantle boundary, where silicate perovskites represent the main mineral phases (Murakami *et al.*, 2012 ▶). Interestingly, part of this depth corresponds to the thermodynamical conditions accessible to conventional HP apparatus (piston cylinder, belt apparatus, multi anvil, diamond anvil) used for the HP/HT synthesis and characterization reported in the present paper.

Thus, it is not surprising that the section of this work on HP/HT synthesis of new materials eventually exhibiting MF properties deals mainly with perovskite-based compounds.

As already pointed out, measurement of the properties of MFs, and in particular the direct magneto-electric coupling, is not trivial, even on high quality samples available in large quantities. This is usually not the case for HP synthesized metastable compounds, where the MF properties are generally detected (or hypothesized) in an indirect way, when the measurement (or the calculation) of the magnetic and ferroelectric transitions occur simultaneously.

### 2.1. HP/HT synthesis of simple perovskite bulk MF compounds   

The ‘simple’ perovskite structure (*AB*O_3_) consists of corner-sharing *B*O_6_ octahedra, with *B* ions (usually magnetic, such as Mn or Fe) in the center of the octahedral site (coordination number: 6) and *A* ions at the center of a cube formed by eight *B*O_6_ octahedra (coordination number: 12). The most common structural distortion derives from the mismatch of *A*—O and *B*—O bond distances, the Jahn–Teller (JT) distortions (elongation/compression) and the buckling (variation of the *B*—O—O angle) of the *B*O_6_ octahedra. The application of an external pressure enhances the ability of the perovskite to accommodate different ionic sizes, vacancies and the above-mentioned structural distortions.

For example, in the manganites (*R*MnO_3_) two crystal phases, hexagonal and ortho­rhombic, exist at ambient pressure. The ortho­rhombic structure (*Pbnm*) is stable for large *R* ions (La, Pr, Nd, Tb and Dy), while small *R* ions (Ho, Er, Yb, Lu, In and Sc) adopt the hexagonal structure (*P*6_3_ *cm*, at room temperature). However, orthorhombic *R*MnO_3_ with small *R* ions, such as Ho and Lu, can be synthesized as metastable perovskites by HP/HT synthesis (Wood *et al.*, 1973 ▶; Wu *et al.*, 2014 ▶; Rodgers *et al.*, 2006 ▶).

The simplest method to induce MF is to combine in the same phase a magnetic ion (ferromagnetism) in a non-centrosymmetric structure, enabling the insurgence of ferroelectricity. Ferroelectricity can be inducted by the asymmetrical coordination produced by the stereochemical effect of a ‘lone-pair’ atom such as Bi^3+^ and Pb^2+^, representing frequent ingredients of the magneto-electric MF compounds.

BiFeO_3_ is the prototype material, but it is unusual among the Bi-based perovskites in that it can be made under ambient conditions; most others require HP/HT synthesis. Since Bi_2_O_3_ melts at 824°C at ambient pressure, HP is necessary in most cases to achieve a high-temperature solid state reaction whenever oxides are used as reagents.

BiMnO_3_ is an obvious MF magneto-electric candidate, requiring HP/HT synthesis (*P* = 4 GPa, *T* = 1273 K). BiMnO_3_ deserves a special discussion; in spite of its ‘simple’ perovskite structure, it is a very complex material and we refer to the massive work that has been done to unveil its peculiar structural properties (Belik, Iikubo *et al.*, 2006 ▶), the HP/HT synthesis (*P* = 4 GPa, *T* = 1273 K), the complex phase diagram at ambient and high temperature (Montanari, Righi *et al.*, 2005 ▶; Montanari, Calestani *et al.*, 2005 ▶), and the magnetic and electric properties (Chou *et al.*, 2009 ▶), some of those are also detailed in the second part of this paper on characterization. We emphasize the controversial determination of its MF properties, since a general consensus on the structure and the ferroelectric properties is still lacking (Goian *et al.*, 2012 ▶).

The search for polar structure in potentially MF Bi-based perovskites has been studied in particular by Belik, who reported a series of Bi*M*O_3_ oxides with *M* = Al, Sc, Ti, V, Cr, Mn, Co, Ni, Cu, Ga, In and Rh (Belik, 2012 ▶). He also investigated the solid solutions of BiGa_*x*_ *M*_1−*x*_O_3_ (*M* = Cr, Mn and Fe) prepared at *P* = 6 GPa and *T* = 1700 K (*M* = Cr and Fe) and 1300 K (*M* = Mn) resulting in the formation of a large family of polar materials with *Cm* and *R*3*c* symmetries. Samples with *Cm* symmetry have structures similar to PbVO_3_ and BiCoO_3_, while *R*3*c* symmetry compounds are isostructural with BiFeO_3_ and have comparable calculated spontaneous polarization (58 µC cm^−2^ for BiGa_0.4_Cr_0.6_O_3_). Noticeable calculated polarizations of 116 µC cm^−2^ and 102 µC cm^−2^ are reported for BiGa_0.4_Fe_0.6_O_3_ and BiGa_0.3_Mn_0.3_O_3_, respectively. On the contrary, the HP synthesized BiMnO_3_-type BiScO_3_ (*P* = 6 GPa, *T* = 1413 K) crystallizes in the space group *C*2/*c* (centrosymmetric and therefore non-ferroelectric) (Belik, Iikubo *et al.*, 2006 ▶).

To provide further evidence of the complex and intriguing behavior of BiMnO_3_, recently Chakrabartty *et al.* reported a photovoltaic effect exploiting its ferroelectricity (*i.e.* the photo-carrier separated by the electrical dipole rather than in a *p*–*n* junction as in a conventional solar cell); an external solar power conversion efficiency of ∼ 0.1% was achieved in Bi–Mn–O thin films grown onto an Nb-doped SrTiO_3_ single-crystal substrate by pulse laser deposition (Chakrabartty *et al.*, 2014 ▶).

Ferroelectric behavior associated with lattice distortion is found in single crystals of perovskite-type YMnO_3_ obtained under HP quasi-hydrothermal conditions (*P* = 5.5 GPa) (Ishiwata *et al.*, 2011 ▶). Polarization along the *ab*-plane was observed, accompanied by the discontinuous jump of the dielectric constant at *T*_N_ = 42 K, below which *P* shows a stepwise increase reaching *ca* 0.22 µC cm^−2^ at 2 K, larger than the value for the polycrystalline sample (Taguchi *et al.*, 2012 ▶).

By combining dielectric, specific heat and magnetization measurements and high-resolution neutron powder diffraction, Ye *et al.* (2007 ▶) investigated the thermodynamic and magnetic and structural properties of the metastable ortho­rhombic perovskite ErMnO_3_ prepared at *P* = 3.5 GPa and *T* = 1373 K. The system shows *T*_N_ = 42 K and a corresponding sudden increase of the dielectric constant.

The monoclinic (*P*2_1_/*n*) ScMnO_3_ perovskite was synthesized by Chen *et al.* (2013 ▶) from the hexagonal phase at *P* = 12.5 GPa and *T* = 1373 K. Although indirect evidence of MF properties have already been obtained, the monoclinic phase shows a similar structure to the orthorhombic Ho and Lu analogues, stimulating further studies on possibly coupled electrical polarization and magnetism.

Pb^2+^ lone-pair asymmetrical coordination was exploited by Shpanchenko *et al.* (2004 ▶) to search for multiferroism in PbVO_3_ synthesized at *P* = 4–6 GPa and *T* = 973–1023 K. The non-centrosymmetrical tetragonal *P*4*mm* structure of corner-shared VO_5_ pyramids rather than octahedra due to a strong tetragonal distortion is found and diffraction data suggest a large ferroelectric polarization above 100 µC cm^−2^ (Belik *et al.*, 2005 ▶). Tetragonal perovskite PbMnO_3_ was obtained by treating the hexagonal perovskite phase at *P* = 15 GPa and *T* = 1273 K (Oka *et al.*, 2009 ▶), but surprisingly the structural analysis suggested that PbMnO_3_ crystallizes in the centrosymmetric space group *P*4/*mmm*, unlike PbVO_3_ and PbTiO_3_ (Chaudhari & Bichile, 2013 ▶), with an antiferromagnetic ordering at *T*_N_ = 20 K.

Varga *et al.* (2009 ▶) reported the HP/HT synthesis of FeTiO_3_ (*P* = 18 GPa, *T* = 1473 K), isostructural with acentric LiNbO_3_. Piezoresponse force microscopy and magnetometry demonstrate the coexistence of ferroelectricity and (weak) ferromagnetism below *T*_N_ = 110 K.

Other interesting examples are ScCrO_3_ and InCrO_3_, synthesized at *P* = 6 GPa and *T* = 1500 K. They crystallize in the GdFeO_3_-type perovskite structure (space group *Pnma*). Antiferromagnetic transitions occur at *T*_N_ = 73 K in ScCrO_3_ and 93 K in InCrO_3_ and dielectric anomalies were observed in both compounds at *T*_N_, indicating magneto-electric coupling, contrary to YCrO_3_ (Belik, Matsushita *et al.*, 2012 ▶).

Aimi *et al.* (2011 ▶) studied the correlation between structure, magnetic and dielectric properties in Mn*M*O_3_ (*M* = Ti, Sn) synthesized in HP/HT conditions (*P* = 7 GPa, *T* = 973 and 1073 K, respectively); both the compounds possess a polar LiNbO_3_-type structure at room temperature. Weak ferromagnetism due to canted antiferromagnetic interactions was observed at *T*_N_ = 25 K for MnTiO_3_ and at *T*_N_ = 50 K for MnSnO_3_, with anomalies in the dielectric permittivity indicating the correlation between magnetic and dielectric properties.

A similar structure was obtained for PbNiO_3_ by Inaguma *et al.* (2011 ▶) at *P* = 3 GPa and *T* = 1073 K. The obtained perovskite-type phase crystallizes in an orthorhombic GdFeO_3_-type structure (space group *Pnma*) that irreversibly transforms to a LiNbO_3_-type phase with an acentric space group *R*3*c* by heat treatment at ambient pressure. The magnetic susceptibility measurement shows that PbNiO_3_ undergoes an antiferromagnetic transition with *T*_N_ = 205 K.

### 2.2. HP/HT synthesis of double perovskite bulk MF compounds   

This paragraph covers the HP/HT synthesis of ‘double’ perovskite MF oxides with the general formula *A*_2_ *BB*′O_6_. The presence of two different magnetic cations on the *B*-site may lead to different types of interactions, favoring an increase in the magnetic ordering temperature and the enhancement of the properties.

Many combinations of ions bringing either magnetic or electric properties have been attempted in the last few years; Shimakawa *et al.* (2011 ▶) reported that bulk Bi_2_NiMnO_6_ (monoclinic structure, space group *C*2, synthesized at *P* = 6 GPa, *T* = 1073 K) exhibits ferromagnetic (*T*_c_ = 485 K) and ferroelectric properties (calculated *P* = 20 µC cm^−2^). Bi_2_FeMnO_6_, a potential MF bulk sample, was obtained by Delmonte *et al.* (2013 ▶) at *P* = 6 GPa at *T* = 1373 K, displaying complex magnetic behavior (magnetic transitions at *T* = 420 and 288 K) and a temperature-induced magnetization reversal. The crystal structure is orthorhombic (space group *Pnam*), with no cation order on the *B* site; anomalies in the thermal dependence of the lattice parameters are observed at the magnetic ordering at *T* = 288 K, indicating a spin-lattice coupling and possible magneto-electric coupling. Cationic disorder of the Bi-based double perovskite is also observed in Bi_2_FeCrO_6_, a recently proposed candidate MF, prepared in bulk form by HP/HT (Suchomel *et al.*, 2007 ▶).

In contrast, a significant degree of ordering of Mn^4+^ and Ni^2+^ ions was observed in In_2_NiMnO_6_, prepared at *P* = 6 GPa and *T* = 1600 K, characterized by a monoclinic structure (space group *P*2_1_/*n*) and magnetic *T*_N_ = 26 K. In spite of the interesting magnetic behavior (a field-induced antiferromagnetic–ferromagnetic transition at low *T*), no magneto-electric coupling has been detected (Yi *et al.*, 2013 ▶). Liu *et al.* (2014 ▶) synthesized high-purity crystals of Y_2_FeMnO_6_ and Y_2_CrMnO_6_, crystallizing in the orthorhombic space group *Pnma*, using a flux method under pressure (*P* = 6 GPa, *T* = 1573 K). An antiferromagnetic transition occurs at *T*_N_ = 328 K in Y_2_FeMnO_6_, and a ferrimagnetic one at *T*_N_ = 74 K in Y_2_CrMnO_6_; in both cases, the presence of the dielectric anomaly near *T*_N_ portends a magneto-electric effect.

Other interesting structures have been recently reported; Mathieu *et al.* (2013 ▶) succeeded in the preparation of Mn_2_FeSbO_6_ at *P* = 3 GPa and *T* = 1273 K, a rare example of an antiferromagnetic double perovskite polymorph (the material also crystallizes as ferrimagnetic ilmenite) with the *A* site entirely occupied by Mn and Fe/Sb cationic order on the *B* sites. Theoretical calculations for the perovskite phase suggest a complex magnetic structure, holding an electronic polarization and possible magneto-electric properties. Aimi *et al.* (2014 ▶) reported the synthesis of a novel ferroelectric *A*-site ordered double perovskite CaMnTi_2_O_6_ at *P* = 7 GPa and *T* = 1473 K, with an uncommon polar space group *P*4_2_ *mc* and a ferroelectric–paraelectric order–disorder type phase transition at 630 K. Similar to the structure of CaFeTi_2_O_6_, the *A*-site Ca^2+^ is 10-coordinated while Mn^2+^ shows alternate tetrahedral and square-planar coordination. The square-planar coordination shifts along the *c*-axis and the usual Ti^4+^ ion displacement accounts for its polar structure. *P*–*E* hysteresis measurement demonstrated that CaMnTi_2_O_6_ is ferroelectric with a calculated polarization and an observed remnant polarization of 24 and 3.5 µC cm^−2^, respectively.

Another promising MF compound is lead iron niobate Pb_2_FeNbO_6_, possessing simultaneously ferroelectric properties below *T* ≃ 380 K, antiferromagnetic order at *T*_N_ ≃ 150 K (Levstik *et al.*, 2008 ▶). Single phases of the (1 − *x*)Pb_2_FeNbO_6 − *x*_Pb_2_FeSbO_6_ solid solution were synthesized at 6 GPa and 1400–1500 K (Raevski *et al.*, 2013 ▶).

### 2.3. HP/HT synthesis of complex (quadruple) perovskite bulk MF compounds   

‘Quadruple’ perovkites are *A*-site ordered compounds, derived by the doubling of the conventional *AB*O_3_ axes; their general formula can be written as *AA*′_3_ *B*_4_O_12_ to better visualize the site occupation of the different elements. The structure is based on a three-dimensional network of corner-sharing tilted octahedra, centered on the *B* site (usually occupied by a magnetic element in MF materials) stabilized under high pressure by the presence of a Jahn–Teller atom (Mn^3+^ or Cu^2+^) on the *A*′ site, forming an uncommon square-planar coordination due to large distortion of the *A*-site coordination.

Among the compounds belonging to this family, CaMn_7_O_12_ (or CaMn_3_Mn_4_O_12_) possesses all the requirements for being a good magneto-electric MF compound: (i) a ferroelectric transition temperature (*T*_C_ = 90 K) much higher compared with other magnetic MF manganites, and (ii) high polarization values, 450 µC m^−2^ at 8 K when using a poling field of 7 kV cm^−1^ (Zhang, Dong *et al.*, 2011 ▶). By neutron powder diffraction from CaMn_7_O_12_ single crystals, Johnson *et al.* (2012 ▶) found that the polarization (along the *c*-axis of the rhombohedral cell) reaches the remarkable value of 2870 µC m^−2^ at low temperatures, one of the largest measured values in magnetic MFs (Johnson *et al.*, 2012 ▶). CaMn_7_O_12_ can be synthesized at ambient pressure although, curiously, was first synthesized under high pressure (Bochu *et al.*, 1974 ▶; *P* = 8 GPa, *T* = 1273 K).

Those impressive properties certainly sparked interest in MF materials belonging to this family of complex perovskites.

Following the consolidated strategy based on the Bi^3+^ substitutions, Mezzadri *et al.* (2009 ▶) reported the HP/HT (*P* = 4 GPa, *T* = 1273 K) synthesis of BiMn_7_O_12_ (or BiMn_3_Mn_4_O_12_). As expected, the structural characterization of single-crystal samples shows a distorted and asymmetrical coordination around the Bi atom due to the presence of the 6*s* ^2^ lone pair, resulting in the non-centrosymmetric space group *Im*, leading to a permanent electrical dipole moment and ferroelectric properties. The dielectric constant shows anomalies, matching the antiferromagnetic transition temperatures (*T*_N_ = 22 and 55 K).

By means of neutron diffraction, Gauzzi *et al.* (2013 ▶) found a large uniform modulation of the antiferromagnetic structure of the Mn^3+^ ions; this modulation (absent in the isovalent compound LaMn_7_O_12_) is induced by the internal strain created by the polar Bi^3+^ ion, and accounts for a large magneto-electric coupling. Therefore, the peculiar quadruple perovskite structure preventing the release of the strain provides a new degree of freedom to achieve large magneto-electric couplings.

Locherer *et al.* (2012 ▶) reported the HP/HT synthesis (*P* = 7.5 GPa, *T* = 1173–1273 K) of the isostructural and heterovalent PbMn_7_O_12_ (or Pb(Mn^3+^_3_)(Mn^3+^_3_Mn^4+^)O_12_ to appreciate the different mixed-valence occupation of Mn on the *B*-site); as for BiMn_7_O_12_, the basic mechanism supporting the FE is supposed to be the stereochemical effect of the 6*s* ^2^ lone pair of Pb^2+^ that induces a permanent electric dipole but, surprisingly, PbMn_7_O_12_ crystallizes in a centrosymmetric structure (space group: 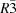). However, a sizeable coupling of magnetic, electrical and dielectric properties at *T*_N_ = 68 K indicates a MF behavior.

This family of compounds is not fully explored yet; new promising properties are awaited, possibly also in non-‘lone-pair’ materials, for their complex structure leading to unusual magnetic properties, such as in LaMn_7_O_12_ (Prodi *et al.*, 2009 ▶) and isostructural samples with decoupling of the magnetic contribution of the *A*′–*B* sites, as in LaMn_3_Cr_4_O_12_ and LaMn_3_Ti_4_O_12_ (Long *et al.*, 2009 ▶).

### 2.4. HP/HT synthesis of distorted-perovskite bulk MF compounds   

Among the numerous structures derived from various distortions of the perovskite structure, iron fluorides with tetragonal tungsten bronze (TTB) structure, having the general formula K_*x*_Fe_*x*_^2+^Fe^3+^_1−*x*_F_3_, deserve a special mention. The structure can be described as a perovskite-like network of Fe^2+^O_6_ octahedra surrounded by pentagonal non-perovskite sites. K_0.6_Fe_0.6_^2+^Fe^3+^_0.4_F_3_ single crystals have been grown by hydrothermal synthesis (*P* = 1.3 Kbar, *T* = 950 K) and below magnetic transitions (*T*_N_ = 118 K) the system, characterized by an ordered arrangement of Fe^2+^/Fe^3+^, is suggested to be a rare example of complete MF, being simultaneously ferrimagnetic, ferroelectric and ferroelastic (Mezzadri *et al.*, 2008 ▶). In this case, the main role of the (limited) applied pressure is to reach the conditions suitable for hydrothermal synthesis.

A selection of perovskite-based materials, their structural properties, HP/HT syntheses conditions and the corresponding magnetic (*T*_N_, *T*_C_) and electric (polarization) transitions is summarized in Table 1 ▶.

Due to unavoidable limitations of the HP/HT syntheses, such as the scarce amount of obtainable material, alternative methods have been developed to mimic the effect of the external pressure: thin film epitaxial stabilization and chemical substitutions.

### 2.5. Beyond HP/HT synthesis of MF materials: thin film epitaxial stabilization   

This approach, desirable for device fabrication, implies the deposition of the single phase (*i.e.* non-hetero-structures formed by a multilayers architecture) in the form of a thin film on strained substrates. Interesting results have been achieved for several (potential) magneto-electric MF materials as a viable alternative to HP/HT synthesis to obtain Bi-based perovskites, such as BiMnO_3_ (Moreira dos Santos *et al.*, 2004 ▶; Sharan *et al.*, 2004 ▶; Eerenstein *et al.*, 2005 ▶), BiAlO_3_ and BiGaO_3_ (Belik, Wuernisha *et al.*, 2006 ▶) on different substrates.

Analogously, fully epitaxial thin films of PbVO_3_ were deposited by a pulsed laser deposition on a number of single-crystal substrates including NdGaO_3_ (100) (Kumar *et al.*, 2007 ▶) and LaAlO_3_ (001) (Oh *et al.*, 2014 ▶). PbVO_3_ has been proposed as a good candidate to be a magneto-electric MF compound, having an antiferromagnetic ordering (*T*_N_ ≃ 200 K) and a ferroelectric polarization as large as 152 µC cm^−2^.

Concerning the double perovskite structure, the tendency is toward MF hetero-structures, that is the combination of layers with different functionalities, by using sophisticated deposition techniques enabling the precise control of the interfaces as detailed in several topical review papers (Shpanchenko *et al.*, 2004 ▶; Ramesh & Spaldin, 2007 ▶; Martin *et al.*, 2008 ▶; Vaz, 2012 ▶).

To the best of our knowledge no MF properties have been reported for the quadruple-perovskite thin film, although for NdMn_7_O_12_, that can be synthesized as bulk at 8 GPa and 1000°C, 10 nm-thick metastable thin film has been successfully grown on Nd_1−*x*_MnO_3_ substrate by the pulsed laser method or by injection MOCVD (Bosak *et al.*, 2000 ▶; Prellier *et al.*, 2001 ▶; Gorbenko *et al.*, 2002 ▶) and the isostructural CaCu_3_Ti_4_O_12_ can be grown onto Pt/Ti/SiO2/Si substrates using a pulsed-laser (Fang & Shen, 2003 ▶).

### 2.6. Beyond HP/HT synthesis of MF materials: ‘chemical’ pressure   

This approach exploits the well known effect of the chemical substitution of atoms with different sizes, mimicking the application of external pressure [see Moritomo *et al.*, 1997 ▶, for a comparison between chemical (internal) pressure and mechanical (external) pressure on perovskite manganites].

To mention a relevant (but not comprehensive) example, Catalan *et al.* (2009 ▶) reported the ambient pressure synthesis of bulk Ca-doped BiFeO_3_ ceramics, for which *T*_N_ increases with Ca concentration (0.66 K per 1% Ca molar). The smaller ionic size of Ca compared with Bi results in a contraction of the lattice, simulating the external pressure at a rate of 1% Ca = 0.3 GPa. Thus, hydrostatic pressure increases the magnetic transition temperature of BiFeO_3_ of 2.2 K GPa^−1^ and the interesting conclusion is that pressure, either chemical or mechanical, may be used to enhance the magneto-electric coupling in MF materials.

The same principle can be applied to thin films; Izquierdo *et al.* (2014 ▶) combined the epitaxial strain and the chemical pressure to modify the magnetic response of Al-doped TbMnO_3_ films grown under compressive/tensile strain using (001)-oriented SrTiO_3_ and MgO substrates by RF-sputtering. The chemical pressure generated by Al doping, together with the substrate-induced strain, modify the subtle competition between magnetic interactions in the system (the films show a weak ferromagnetic phase coexisting with the expected ‘bulk’ antiferromagnetic phase), hence an additional degree of freedom to control the magnetic ordering can be provided, for example, by varying the film thickness and/or using other substrates.

### 2.7. Effect of HP on electrical and magnetic measurements   

Besides the HP/HT synthesis, the application of external pressure often plays an important role in the magneto-electrical characterization even on thermodynamically stable compounds. This effect is clearly reported by Retuerto *et al.* (2009 ▶), comparing the DC-magnetic susceptibility *versus T* and *H* of the double perovskite Sr_2_FeMoO_6_ synthesized at ambient pressure and at 2 GPa, the latter displays a higher saturation magnetization and a sharper ferromagnetic transition at *T*_C_ as high as 430 K, thanks to the HP-induced cationic ordering. Although Sr_2_FeMoO_6_, contrary to the isostructural compounds reported in §2.2, does not belong to the family of MF materials, it indicates that HP is an important tool to better probe their magnetic and electric properties.

Relevant examples include enhancements in the polarization and magnetic properties in BiFeO_3_ ceramic prepared by HP/HT synthesis (Su *et al.*, 2007 ▶), the improved multiferroic properties in bulk *R*Mn_2_O_5_ (*R* = Tb, Dy, Ho) (dela Cruz *et al.*, 2007 ▶) or Ru-doped BiFeO_3_ thin films (Feng *et al.*, 2010 ▶), or other phenomena such as the HP-induced polarization reversal in multiferroic YMn_2_O_5_ (Chaudhury, dela Cruz *et al.*, 2008 ▶), the HP-induced spin-liquid phase of bulk YMnO_3_ (Kozlenko *et al.*, 2008 ▶) or the HP-induced increase in the microwave absorption properties of bulk BiFeO_3_ (Wen *et al.*, 2010 ▶).

## 3. *In situ* investigations of MF materials at HP   

Pressure is an ideal tool to manipulate the electronic and magnetic structure and the atomic arrangement of a material (Hemley & Ashcroft, 1998 ▶; Hemley *et al.*, 2009 ▶; Schilling, 2000 ▶; Struzhkin *et al.*, 2000 ▶). Although some HP phases can be quenched to ambient conditions and be recovered for detailed experimental characterization (as reviewed in §2 of this article), the majority of pressure-induced structural, electronic and magnetic phase transitions are reversible. Therefore, it is paramount to investigate the high-pressure behavior of materials *in situ*. The advances in HP technology over the past 50 years at large scale facilities (*e.g.* synchrotron radiation facilities and neutron sources) and in the laboratory (Bassett, 2009 ▶; Liebermann, 2011 ▶) have provided us with a large number of experimental tools to investigate the response of materials *in situ* at high pressure and variable temperature.

### 3.1. Multiferroic materials with perovskite structure   

The vast majority of investigations at high pressure have been conducted on compounds with perovskite-type structure or variations hereof (we stressed its pivotal role in the synthesis of MF materials in the previous section). The geometrical features of the perovskite structures can be manipulated by the application of high pressure; since there is a direct link between the atomic structure and the magneto-electric properties, dramatic changes in the multiferroic behavior can be expected at high pressure.

#### 3.1.1. BiFeO_3_ a prototype multiferroic material   

As already mentioned, BiFeO_3_ is a prototype MF material with perov­skite structure that shows magnetic and ferroelectric behavior with a strong polarization at *T* = 300 K (Teague *et al.*, 1970 ▶; Wang *et al.*, 2003 ▶; Catalan *et al.*, 2009 ▶; Shvartsman *et al.*, 2007 ▶; Lebeugle *et al.*, 2007 ▶). Over the past years, BiFeO_3_ has developed into the most popular model system for experimental and theoretical investigations of MF materials at HP. The pressure-induced changes in BiFeO_3_ have been experimentally investigated by Raman spectroscopy (Haumont *et al.*, 2006 ▶; Yang *et al.*, 2009 ▶; Guennou, Bouvier, Chen *et al.*, 2011 ▶), X-ray powder and single-crystal diffraction (Belik *et al.*, 2009*a* ▶; Haumont *et al.*, 2009 ▶; Zhu *et al.*, 2010 ▶; Zhang *et al.*, 2013 ▶; Guennou, Bouvier, Chen *et al.*, 2011 ▶; Guennou, Bouvier, Haumont *et al.*, 2011 ▶; Mishra *et al.*, 2013 ▶), neutron powder diffraction (Kozlenko *et al.*, 2011 ▶), optical absorption spectroscopy (Gomez-Salces *et al.*, 2012 ▶; Gavriliuk *et al.*, 2008 ▶), resistivity measurements (Gavriliuk *et al.*, 2008 ▶), Nuclear Forward Scattering (NFS) (Gavriliuk *et al.*, 2005 ▶, 2008 ▶) and X-ray Emission Spectroscopy (XES) (Gavriliuk *et al.*, 2008 ▶) as well as theoretically through first-principles calculations (Gonzalez-Vazquez & Iniguez, 2009 ▶; Shang *et al.*, 2009 ▶; Feng *et al.*, 2013 ▶; Qiang *et al.*, 2013 ▶). BiFeO_3_ displays a complex response to high pressure with a number of structural phase transitions in the pressure range up to 60 GPa (Belik *et al.*, 2009*a* ▶; Haumont *et al.*, 2009 ▶; Zhu *et al.*, 2010 ▶; Guennou, Bouvier, Chen *et al.*, 2011 ▶; Kozlenko *et al.*, 2011 ▶; Mishra *et al.*, 2013 ▶). However, the number of phase transitions, the atomic structure and the symmetry of the HP phases remain controversial. Gavriliuk *et al.* (2008 ▶) observed no structural phase transition in the pressure range to 60 GPa. Zhu *et al.* (2010 ▶) proposed a structural phase transition at about 10 GPa, however, based on the low-resolution diffraction data no unit cell or space group were proposed for the HP phase. Kozlenko *et al.* (2011 ▶) proposed a phase transition at 3 GPa from *R*3*c* to an orthorhombic phase with the space group *Pbam* from neutron powder diffraction data. Haumont *et al.* (2009 ▶) reported two phase transitions at *P* ≃ 3.5 and 10 GPa and proposed a *R*3*c* → *C*2/*m* → *Pnma* phase sequence. Mishra *et al.* (2013 ▶) observed two phase transitions at *P* = 4.1 and 11 GPa with a proposed phase sequence of *R*3*c* → *P*222_1_ → *Pnma*. Two phase transitions at *P* = 4 and 7 GPa on compression have been observed by Belik *et al.* (2009*a* ▶) and an additional phase transition on decompression, stable in a narrow pressure range between 3.4 to 4.9 GPa. The resulting phase transition sequence is the following *R*3*c* → *Pbam* → *Ibam* → *Cmmm* proposing three different low-pressure phases with orthorhombic symmetry (Belik *et al.*, 2009*a* ▶). Guennou, Bouvier, Chen *et al.* (2011 ▶) conducted X-ray single-crystal diffraction experiments up to pressures of 60 GPa, reporting six structural phase transitions at *P* = 4, 5, 7, 11 and 38 GPa, respectively. The proposed phase sequence is *R*3*c* → O-I → O-II → O-III → *Pnma* → *Pnmm* → *Cmcm*. The limited reciprocal space coverage of the HP single-crystal diffraction data did not allow an unambiguous identification of the structure models or space groups of the low-pressure orthorhombic O-I, O-II and O-III phases. Gavriliuk *et al.* (2008 ▶) conducted XES, NFS and resistivity measurements up to *P* = 55 GPa. The results from the XES and NSF measurements suggest a spin crossover from high spin (HS) to low spin (LS) of Fe^3+^ in the region between 45 to 55 GPa and a reversible insulator to metal Mott transition above 55 GPa. The authors suggest that the insulator to metal transition is driven by the HS–LS transition of the Fe^3+^, by changing the effective correlation energy below the threshold for the insulator to metal transition. First-principles calculations (Gonzalez-Vazquez & Iniguez, 2009 ▶) confirm the HS–LS transition and the very complex structural-electronic magnetic interplay during the transformations observed experimentally by Gavriliuk *et al.* (2008 ▶). Furthermore, an additional transition to a metallic non-spin phase at pressures above 70 GPa was proposed (Gonzalez-Vazquez & Iniguez, 2009 ▶).

Haumont *et al.* (2006 ▶) and Guennou, Bouvier, Haumont *et al.* (2011 ▶) find in their experimental results that stress and non-hydrostatic pressure conditions can not only lead to a significant shift in transition pressures, but also to stabilization of new phases. Guennou, Bouvier, Haumont *et al.* (2011 ▶) identified a new monoclinic phase at *P* ≃ 8 GPa under non-hydrostatic pressure conditions. In order to further understand the structural richness of BiFeO_3_, Dieguez *et al.* (2011 ▶) conducted a systematic investigation of the stability of potential BiFeO_3_ phases using first-principle methods. The results show a large number of metastable phases, which might explain the observation of a large number of HP and different phases reported.

The influence of impurities and defects on the HP behavior of BiFeO_3_ has been investigated by Chen *et al.* (2012 ▶). Ba-doped BiFeO_3_ (Bi_1−*x*_Ba_*x*_FeO_3−0.5*x*_) has been compressed to 18.7 GPa and significant changes in the compression behavior, the phase transition pressure and the phase sequence have been reported compared with pure BiFeO_3_, observing a similar phase sequence, *R*3*c* → *C*2/*m* → *Pnma*, as Haumont *et al.* (2009 ▶).

#### 3.1.2. Rare-earth manganite, *R*MnO_3_ (*R* = Gd, Tb, Dy, Ho, La, Y, Lu, Tm, Sc, Dy)   

Rare-earth manganites show a complex correlation between structural, electric and magnetic properties, which can be manipulated by exchanging the rare-earth ions. As of now, MF properties have only been reported for a subset of the rare-earth manganites and only a few have been characterized at high pressure.

The HP behavior of TbMnO_3_ was investigated by X-ray diffraction and X-ray absorption spectroscopy (XAS). A continuous decrease in the Jahn–Teller distortion as well as a decrease in the tilt angles of the MnO_6_ octahedra was observed with increasing pressure. XAS measurements show a shift of the Mn *K*-edge to higher energies while the pre-edge feature shifts to lower energies with pressure. Chen *et al.* (2009 ▶) interpret this as a broadening of the electronic band width of the *e_g_*↑ orbitals in TbMnO_3_.

Chou *et al.* (2013 ▶) investigated the response of the local and electronic structure of DyMnO_3_ to pressure by Raman spectroscopy, XAS and XES measurements. A decrease of the Jahn–Teller distortion of the MnO_6_ octahedra in combination with the gradual breakdown of the high-spin magnetism is detected at *P* = 32 GPa, suggesting a potential HS to LS transition above 32 GPa. No structural phase transitions were detected in the investigated pressure range.

GdMnO_3_ has been investigated to pressures of 63 GPa by X-ray diffraction (Lin, Zhang *et al.*, 2012 ▶) and to pressures of 53 GPa by X-ray diffraction and Raman spectroscopy measurements (Oliveira *et al.*, 2012 ▶). A reversible first-order phase transition has been observed at *P* ≃ 50 GPa. Based on the diffraction data Lin, Zhang *et al.* (2012 ▶) interpreted the transition as an isostructural orthorhombic to orthorhombic transition. From X-ray diffraction and Raman spectroscopy measurements, Oliveira *et al.* (2012 ▶) conclude that it is an insulator to metal transition with a change in symmetry from orthorhombic (*Pnma*) to cubic (*P*2_1_3).

Kozlenko and co-workers investigated the crystal and magnetic structure of hexagonal YMnO_3_ (Kozlenko *et al.*, 2008 ▶; Kozlenko, Kichanov*, et al.*, 2010 ▶) and LuMnO_3_ (Kozlenko, Kichanov *et al.*, 2010 ▶) by simultaneous high-pressure and low-temperature neutron powder diffraction. The reduction of the in-plane splitting of the Mn—O bond with pressure has been identified as the potential reason for the observed enhanced spin fluctuations with increasing pressure in both compounds. Kozlenko, Kichanov *et al.* (2010 ▶) concluded that the reduction in Mn—O bond splitting with pressure implies a decrease in the magneto-elastic coupling strength.

Wang *et al.* (2010 ▶) investigated the high-pressure behavior of hexagonal TmMnO_3_ by X-ray powder diffraction experiments up to *P* = 28.6 GPa. A hexagonal to orthorhombic transition was detected at 10.2 GPa.

ErMnO_3_ crystallizes in the hexagonal space group *P*6_3_ *cm*. At *P* ≃ 20 GPa the structure undergoes an irreversible first-order transition to the orthorhombic phase with space group *Pbnm* (Lin, Liu *et al.*, 2012 ▶).

The structural stability of *R*MnO_3_ (*R* = Y, Ho, Lu) at HP has been investigated by X-ray diffraction, synchrotron IR spectroscopy, XAS and *ab-initio* quantum mechanical calculations (Gao *et al.*, 2011 ▶). YMnO_3_, HoMnO_3_ and LuMnO_3_ remain hexagonal up to *P* ≃ 20 GPa. Above 20 GPa the onset of a structural phase transition to orthorhombic symmetry has been observed. The IR measurements show that the O atoms are the most sensitive to pressure, with vibration modes mainly confined to the *ab* plane (Gao *et al.*, 2011 ▶).

#### 3.1.3. BiMnO_3_   

A lot of work has been carried out on BiMnO_3_ and HP characterization. The unit cell changes in BiMnO_3_ with pressure were investigated by energy dispersive diffraction in the pressure range from ambient pressure to 27 GPa (Chi *et al.*, 2008 ▶). The measurements showed no evidence of a structural phase transition. The DC magnetization was measured at pressures up to 1.6 GPa by Chou *et al.* (2008 ▶). *T*_C_ = 100 K at ambient pressure and the signal associated with ferromagnetic behavior decreases with pressure and disappears at *P* = 1.31 GPa. A second peak in the susceptibility has been detected at 1.17 GPa and has been interpreted as an onset of a structural phase transition (Chou *et al.*, 2008 ▶). These measurements were followed up by magnetic hysteresis and AC susceptibility to high pressure and low temperature (Chou *et al.*, 2009 ▶). Three magnetic phase transitions have been detected at ambient pressure and 98 K, 0.87 GPa and 93 K, and 72.5 K and 0.87 GPa. The ambient pressure and 100 K transition can be characterized as a long-range ferromagnetic transition, being suppressed at high pressure and therefore not observed. The transition is accompanied by an anomaly below 90 K, which is attributed to a spin-glass behavior. The second transition is attributed to a long-range soft ferromagnetic to a canted state, while the third is characterized as a canted antiferromagnetic transition. Chou *et al.* (2009 ▶) suggest that both the canted ferromagnetic and the canted antiferromagnetic transition are caused by the structural phase change from *C*2/*c* to *P*2_1_/*c*, which has been observed by Belik *et al.* (2009*b* ▶). This demonstrates the complex interplay between lattice distortion and spin configuration in multiferroic material. Belik *et al.* (2009*b* ▶) reported two structural phase transitions at 0.9 and 8 GPa. The phase transition at 0.9 GPa has been characterized as a *C*2/*c* → *P*2_1_/*c* transition, however, the structure of the new monoclinic HP phase could not be refined. At 8 GPa BiMnO_3_ transforms to the non-ferroelectric GdFeO_3_-type structure with *Pnma* symmetry. The GdFeO_3_-type structure exhibits a strong Jahn–Teller distortion of the MnO_6_ octahedra and long-range *d*(3*y* ^2^ − *r* ^2^) *e_g_* orbital ordering (Belik *et al.*, 2009*b* ▶). Kozlenko, Belik *et al.* (2010 ▶) conducted high-pressure neutron powder diffraction measurements on BiMnO_3_ to 10 GPa and simultaneous high-pressure and low-temperature neutron diffraction experiments in the pressure region up to 2 GPa. The ferromagnetic ground state is suppressed at moderate pressures of 1 GPa at 90 K with a transition to an antiferromagnetic state with a propagation vector of *k* = (½ ½ ½) at 90 K. This change in magnetic state is accompanied by a monoclinic to monoclinic structural phase transition. The FM and AFM states coexist below pressures of 2 GPa (Kozlenko, Belik *et al.*, 2010 ▶). Mei *et al.* (2010 ▶) explored the structural and elastic properties of BiMnO_3_ by first principles calculations using LDA+U and GGA+U formalisms. The energies for the monoclinic (*C*2/*c*) phases and the orthorhombic (*Pnma*) phase were calculated as a function of pressure. The calculations, in contrast to the experimental results by Belik *et al.* (2009*b* ▶), show that the orthorhombic phase is the stable structure at ambient pressure. A monoclinic to monoclinic phase transition is predicted at *P* = 10 GPa induced by a magnetic to volume instability in BiMnO_3_. Recently, Kozlenko *et al.* (2014 ▶) determined the phase diagram of BiMnO_3_ to a pressure of 50 GPa and investigated the phase relationships as a function of temperature and pressure in the range *T* = 300–900 K from ambient pressure to 4 GPa using X-ray diffraction and Raman spectroscopy. Two new high-pressure phases with *C*2/*m* symmetry, but distinctly different structural parameters, have been identified at moderate pressures and temperatures, as had been previously suggested based on the results from first-principles calculations (Mei *et al.*, 2010 ▶). The transition to the high temperature orthorhombic phase is suppressed to room temperature at 8 GPa, consistent with reports from Belik *et al.* (2009*b* ▶). Above 20 GPa BiMnO_3_ undergoes a phase transition from *Pnma* → *Imma*, leading to a suppression of the long-range *d*(3*y* ^2^ − *r* ^2^) *e_g_* orbital ordering.

#### 3.1.4. Cobaltite perovskites, *A*CoO_3_   

Ming *et al.* (2009 ▶) studied the structural stability, and the magnetic and electronic properties of BiCoO_3_ by first-principles calculations up to 30 GPa. The calculations reproduce the C-type antiferromagnetic structure as a ground state. At *P* = 4 GPa a first-order isostructural transition is observed, accompanied by a HS–LS spin-crossover of Co^3+^ with an insulator to semi-metal transition. The authors conclude that in contrast to the excluded models for insulator to metal transitions, the spin-crossover effect at HP is the driving mechanism for the transition in BiCoO_3_. Oka *et al.* (2010 ▶) investigated the HP behavior of BiCoO_3_ by synchrotron and neutron powder diffraction and X-ray emission spectroscopy. A first-order tetragonal, PbTiO_3_-type, to orthorhombic, GdFeO_3_-type, phase transition has been observed at *P* = 3 GPa, in contrast to the proposed isostructural tetragonal to tetragonal transition by Ming *et al.* (2009 ▶). The transition is accompanied by a HS to LS transition with an intermediate spin state present at the transition as determined from XES data.

#### 3.1.5. PbNiO_3_   

As already mentioned, two synthesized polymorphs of PbNiO_3_ can be obtained by HP/HT synthesis, with a perovskite-type and a LiNbO_3_-type structure (Inaguma *et al.*, 2011 ▶). The unit-cell volume for LiNbO_3_ and the perovskite-type polymorph suggest that the perovskite type might be a high-pressure polymorph of the LiNbO_3_-type PbNiO_3_. The LiNbO_3_-type PbNiO_3_ undergoes a pressure-induced phase transition to a GdFeO_3_-type structure, space group *Pnma*, at about 3 GPa. In both polytypes the magnetic interaction of the Ni^2+^ is antiferromagnetically dominated by the Ni—O distances rather than the Ni—O—Ni angles. XPS measurements suggest Pb^4+^, Ni^2+^ valence states for both polymorphs. Hao *et al.* (2014 ▶) performed first principles calculation using the GGA, GGA+U and Heyd-Scuseria-Ernzerhof (HSE) approaches. The observed pressure-induced phase transition (Inaguma *et al.*, 2011 ▶) from *R*3*c* to *Pnma* is reproduced, although at a slightly higher transition pressure of about 5 GPa. The orthorhombic HP phase shows a considerable anisotropy of the nearest-neighbor exchange couplings.

#### 3.1.6. BiNiO_3_   

Azuma *et al.* (2007 ▶) investigated the high-pressure behavior of BiNiO_3_ by neutron diffraction experiments in combination with electronic-state calculations using first principles with the LDA+U approach. At 4 GPa a pressure-induced phase transition from the 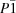 to GdFeO_3_-type structure, with space group *Pbnm*, has been observed. Calculation of the changes in electronic structure with pressure suggest that the pressure-induced phase transition is an insulator to metal transitions which occurs *via* simultaneous melting of the Bi charge disproportion and charge transfer from Ni to Bi.

### 3.2. MF materials with non-perovskite structure   

Recently, the search for MFs has expanded to materials that crystallize in other structure types than the perovskite-type structure and to chemical compositions beyond ‘simple’ oxides such as transition metal oxyhalides, germanates and silicates, transition metal orthotellurides and transition metal chalcogenides. These materials are relatively new and none of them require HP/HT synthesis, but a few have been investigated *in situ* at high pressure.

#### 3.2.1. MnWO_4_   

MnWO_4_ crystallizes in the wolframite structure (space group *P*2/*c* with *Z* = 2) and is ferroelectric showing incommensurate helical spin-density waves (Taniguchi *et al.*, 2006 ▶). The pressure dependence of the dielectric properties up to pressures of 1.8 GPa has been measured by Chaudhury, Yen *et al.* (2008 ▶). Pressure suppresses the ferroelectric polarization in the ferroelectric phase suggesting that the stability of the ferroelectric phase is reduced by increasing the pressure. The structural changes in MnWO_4_ with pressure have been investigated to 8 GPa and the compression mechanism has been derived from X-ray diffraction data (Macavei & Schulz, 1993 ▶). *Ab initio* quantum mechanical calculations (López-Moreno *et al.*, 2009 ▶) confirm the experimentally determined compression behavior of MnWO_4_ (Macavei & Schulz, 1993 ▶) and suggest no structural phase transition in the pressure range from ambient pressure to 31 GPa. The calculated magnetic moment decreases with pressure confirming the results from Chaundhury, Yen *et al.* (2008 ▶). Recently, Dai *et al.* (2013 ▶) determined the pressure and temperature behavior of MnWO_4_ by Raman scattering. Changes in the Raman spectrum at *P* = 17.7 GPa at ambient temperature were interpreted as a monoclinic to triclinic structural phase transition.

#### 3.2.2. CuCrO_2_   

CuCrO_2_ crystallizes in the delafossite-type structure (space group 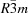) and shows multiferroic properties below *T*_N_ = 24 K, at which the magnetic moments ordered into a proper screw-type spiral structure (Seki *et al.*, 2008 ▶; Kimura *et al.*, 2008 ▶). The Cr^3+^ ions in the structure are arranged in triangular planes, which are stacked along the *c* axis. Spin frustration occurs on the triangular planes, leading to a degree of freedom of spin chirality in the magnetic screw-type spiral structure. The spin chirality allows a relaxation of the spin frustration and therefore has a stabilizing effect on the structure. A *p*–*d* hybridization model can be used to fully describe the ferroelectricity in conjunction with the screw spiral ordering (Arima, 2007 ▶). Aoyama *et al.* (2013 ▶) investigated the evolution of the crystal structure, magnetism and spin-driven ferroelectricity in CuCrO_2_ up to pressures of 10 GPa. The temperature *T*_N_ of the transition to the spin-spiral ferroelectric ordering in CuCrO_2_ increases with pressure. The magnitude of the dielectric anomaly at *T*_N_ is suppressed with increasing pressure and the spontaneous polarization is completely suppressed at about 8 GPa (Aoyama *et al.*, 2013 ▶). Aoyama *et al.* (2013 ▶) attribute the ferroelectric antiferroelectric transitions to a shortening of the Cr-interlayer distances, which leads to an increase in the interlayer exchange integral with elevated pressure. The observed increase in the size of the field for the polarization reversal with pressure can be attributed to a rearrangement in the magneto-electric domains (Aoyama *et al.*, 2013 ▶).

#### 3.2.3. Ba_3_TaFe_3_Si_2_O_14_   

Ba_3_TaFe_3_Si_2_O_14_, a member of the langasite family, crystallizes in the trigonal space group *P*321 with one formula unit per unit cell (Lyubutin *et al.*, 2010 ▶). Gavriliuk *et al.* (2013 ▶) investigated the pressure and temperature behavior of Ba_3_TaFe_3_Si_2_O_14_ up to *P* = 38 GPa and down to *T* = 20 K by Nuclear Forward Scattering (NFS) and Raman spectroscopy. At *P* = 19.5 GPa a first-order pressure-induced phase transition has been observed. The transition is accompanied by a magnetic change with a fourfold increase in Néel temperature *T*_N_ from 27.2 to 120 K (Gavriliuk *et al.*, 2013 ▶). The authors suggest a strong superexchange interaction caused by changes in the bond angles as an explanation for the strong pressure dependence of the Néel temperature.

#### 3.2.4. Mn_2_GeO_4_   

Recently, multiferroic properties have been reported in Mn_2_GeO_4_, a compound that crystallizes in the orthorhombic (space group *Pnma*) olivine-type structure (Creer & Troup, 1970 ▶). At low temperature, Mn_2_GeO_4_ undergoes three antiferromagnetic (AFM) phase transitions at *T*_N1_ = 47 K > AFM1 > *T*_N2_ = 17 K > AFM2 > *T*_N3_ = 5.5 K > AFM3 (White *et al.*, 2012 ▶; Volkov *et al.*, 2013 ▶; Honda *et al.*, 2012 ▶). The transitions are characterized by a change in the magnetic moments of Mn^2+^. The AFM1 phase shows mainly an alignment of the moments along the *a* axis with a slight canting along the *c* axis, making AFM1 a weak ferromagnetic phase. The spins are oriented along the *b* axis in the AFM2 phase. The AFM3 phase exhibits spontaneous magnetization and electric polarization along the *c* axis, characteristic for a MF phase. The ferroelectricity originates from the formation of an incommensurate (IC) spin-spiral structure and the spontaneous magnetization arises from a canting of a commensurate (C) magnetic structure (White *et al.*, 2012 ▶). Honda *et al.* (2014 ▶) investigated the effect of pressure on the magnetism and the multiferroicity of Mn_2_GeO_4_. The transition temperatures of the magnetic transitions change monotonically with increasing pressure. The change in transition temperature *T*_N1_ [*d* *T*_N1_/*d* *p* = 0.046 (3) GPa^−1^] can be described by a classical Heisenberg exchange Hamiltonian model assuming the size of the superexchange interactions scale proportionally to the power −10/3 of the volume (Honda *et al.*, 2014 ▶). At 6 GPa the spin-driven ferroelectricty in the AFM3 phase disappears. High-pressure neutron diffraction experiments observed a shift of the IC magnetic peak towards a C position with increasing pressure (Honda *et al.*, 2014 ▶). Therefore, the suppression of the spin-driven ferroelectricity in the AFM3 phase at 6 GPa is attributed to an IC-C phase transition.

#### 3.2.5. FeTe_2_O_5_Br   

A relatively new class of MF materials is the group of transition metal oxohalides (Choi *et al.*, 2014 ▶). The incorporation of lone-pair cations and halides provides exchange pathways, which lead to magnetic frustration in these materials (Bos, Colin & Palstra, 2008 ▶; Lawes *et al.*, 2003 ▶; Pregelj *et al.*, 2009 ▶; Zaharko *et al.*, 2006 ▶; Zhang, Kremer *et al.*, 2011 ▶). FeTe_2_O_5_Br is not only a magnetically but also a geometrically frustrated system as well. The HP behavior of FeTe_2_O_5_Br has been investigated by Raman and optical absorption spectroscopy up to *P* = 7 GPa (Gnezdilov *et al.*, 2011 ▶; Choi *et al.*, 2014 ▶). Both, the Raman spectroscopy and the optical absorption measurements show evidence of a structural phase transition between 2.12 and 3.04 GPa. The HP phase exhibits an increased uniformity in the bonding forces, compared with the highly anisotropic ambient pressure phase. The anisotropic Fe(3*d*) orbitals start to overlap with the Br(4*s*) and O(2*p*) orbitals, changing from a two-dimensional electronic structure at ambient conditions to a three-dimensional electronic structure at HP.

#### 3.2.6. HgCr_2_S_4_   

HgCr_2_S_4_ belongs to the large group of thiospinels and crystallizes in the space group 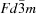 at ambient conditions (Hemberger *et al.*, 2006 ▶; Weber *et al.*, 2006 ▶). The compound exhibits both ferromagnetic and ferroelectric properties together with a pronounced magneto-capacitive coupling with a bond-frustrated magnetic ground state (Hemberger *et al.*, 2006 ▶). The ferroelectricity in the chromium thiospinel family is caused by a dynamic disorder of the Cr^3+^ ions, which induces an off-center shift of the Cr^3+^ ions and a change in symmetry of the local structure to the polar space group 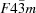 (Gnezdilov *et al.*, 2011 ▶). The HP behavior of HgCr_2_S_4_ has been investigated by a combination of X-ray powder diffraction measurements and band structure calculations based on the Linear-Muffin-Tin Orbital method (Efthimiopoulos *et al.*, 2013 ▶). Two structural phase transitions have been observed at *P* = 20 and 26 GPa, respectively. At 20 GPa HgCr_2_S_4_ undergoes a reversible first-order cubic to tetragonal transition from the space group 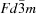 to *I*4_1_/*amd*. The phase transition has an insulator to metal character and is accompanied by a change in the magnetic state to an antiferromagnetic one and the loss of ferroelectric properties. A second-order phase transition to an orthorhombic structure, most likely with *Imma* space group, has been reported at 26 GPa. Based on the diffraction data Efthimiopoulos *et al.* (2013 ▶) proposed a third phase transition at *P* ≃ 37 GPa. However, strong peak overlap between gasket, the ortho­rhombic phase and the potentially new HP phase prevented the detailed characterization of this phase.

## 4. Conclusions   

The search for intrinsic magneto-electric MF compounds with the co-existence and coupling of magnetic and electric order in the same phase remains challenging despite large efforts in the scientific community. Most currently known intrinsic MF only exhibit very small magneto-electric coupling at low temperature, while for technological applications they are required to show large magneto-electric effects at ambient conditions. Therefore, it is paramount to broaden the search for MF materials to new classes of materials and to new synthesis techniques.

The HP/HT synthesis has proven to be a powerful tool for the stabilization of metastable HP phases with MF properties. The majority of these new phases crystallize in the perovskite or perovskite-related structure. The investigation of MF materials *in situ* at high pressure and temperature has provided valuable insight into the coupling between electric, magnetic and structural properties. Especially experiments at non-hydrostatic conditions can in the future lead to the identification of possible high-pressure MF phases that could be stabilized at ambient conditions as highly strained thin films.

Despite the promising results obtained so far, the currently employed high-pressure techniques have intrinsic shortcomings, such as comparatively long synthesis times, relatively small sample sizes and vast parameter space in pressure, temperature and composition that needs to be explored. However, these limitations can be overcome by a concerted effort between theory (*e.g.* structure prediction), which could potentially allow targeted synthesis at pressure and temperature, and the development of new HP synthesis and characterization approaches.

The availability of such new and more sophisticated synthesis and characterization techniques, combined with theoretical approaches, could lead to an enormous increase in our fundamental understanding of MF materials and potentially to widespread technological applications.

## Figures and Tables

**Table 1:** HP/HT synthesis and magneto-electric properties in MF materials with perovskite-based structure

Material	Synthesis conditions	Space group	Magneto-electric properties	Notes
BiMnO_3_	*P* = 4GPa	?	*T*_N_ = 100K	Still controversial!!
	*T* = 1273K	(Belik, Iikubo *et al.*, 2006 ▶; Belik, 2012 ▶; Montanari, Righi *et al.*, 2005 ▶; Montanari, Calestani *et al.*, 2005 ▶; Chou *et al.*, 2009 ▶; Goian *et al.*, 2012 ▶)		Centro-/non-centrosymmetric?
BiGaO_3_	*P* = 6GPa	*Pcca*	*P*> 100Ccm^2^	Calculated *P* for BiGa_1*x*_ *M_x_*O_3_, *M* = Cr, Mn, Fe (space group: *R*3*c*)
	*T* = 1473K	(Belik, Rusakov *et al.*, 2012 ▶)		
YMnO_3_	*P* = 5.5GPa	*P*2_1_ *nb*	*T*_N_ = 42K	Increase of dielectric constant @ *T*_N_
	*T* = 1273K	(Wood *et al.*, 1973 ▶)	*P* = 0.22Ccm^2^	
ErMnO_3_	*P* = 3.5GPa	*Pbnm*	*T*_N_ = 42K	Sudden increase of dielectric constant @ *T*_N_
	*T* = 1373K	(Ye *et al.*, 2007 ▶)		
ScMnO_3_	*P* = 12.5GPa	*P*2_1_/*n*	*T*_N_ = 51K	Not (yet) direct evidence of *M* *E* coupling
	*T* = 1373K	(Chen *et al.*, 2013 ▶)		
PbVO_3_	*P* = 5GPa	*Pnma*	*P* = 100Ccm^2^	VO_5_ pyramids
	*T* = 1023K	(Shpanchenko *et al.*, 2004 ▶)	(Varga *et al.*, 2009 ▶)	
PbMnO_3_	*P* = 5GPa	*P*4/*mmm*	*T*_N_ = 20K	Centrosymmetric structure, non-FE
	*T* = 1023K	(Oka *et al.*, 2009 ▶)		
FeTiO_3_	*P* = 18GPa	*R*3*c*	*T*_N_ = 110K	Evidence of *M* *E* coupling @ *T*_N_
	*T* = 1473K	(Varga *et al.*, 2009 ▶)		
ScCrO_3_	*P* = 6GPa	*Pnma*	*T*_N_ = 73K (SCO)	Anomaly in dielectric constant @ both *T*_N_
InCrO_3_	*T* = 1500K	(Belik, Matsushita *et al.*, 2012 ▶)	*T*_N_ = 93K (ICO)	
MnTiO_3_	*P* = 7GPa	*R*3*c*	*T*_N_ = 25K	Anomaly in dielectric constant @ *T*_N_
	*T* = 973K	(Aimi *et al.*, 2011 ▶)		
MnSnO_3_	*P* = 7GPa	*R*3*c*	*T*_N_ = 50K	Anomaly in dielectric constant @ *T*_N_
	*T* = 1073K	(Aimi *et al.*, 2011 ▶)		
PbNiO_3_	*P* = 3GPa	*R*3*c*	*T*_N_ = 205K	LiNbO_3_-like acentric structure
	*T* = 1073K	(Inaguma *et al.*, 2011 ▶)		
Bi_2_NiMnO_6_	*P* = 6GPa	*C*2	*T*_C_ = 485K	Evidence of *M* *E* coupling
	*T* = 1073K	(Shimakawa *et al.*, 2011 ▶)	*P* = 20Ccm^2^	
Bi_2_FeMnO_6_	*P* = 6GPa	*Pnam*	*T*_N_ = 288K	Reversal magnetization
	*T* = 1373K	(Delmonte *et al.*, 2013 ▶)		Evidence of *M* *E* coupling
In_2_NiMnO_6_	*P* = 6GPa	*P*2_1_/*n*	*T*_N_ = 26K	Complex magnetic behavior
	*T* = 1600K	(Yi *et al.*, 2013 ▶)		No evidence of *M* *E* coupling
Y_2_FeMnO_6_	*P* = 6GPa	*Pnma*	*T*_N_ = 328K	Evidence of *M* *E* coupling dielectric anomaly @ *T*_N_
	*T* = 1573K	(Liu *et al.*, 2014 ▶)		
Y_2_CrMnO_6_	*P* = 6GPa	*Pnma*	*T*_N_ = 74K	Evidence of *M* *E* coupling dielectric anomaly @ *T*_N_
	*T* = 1573K	(Liu *et al.*, 2014 ▶)		
Mn_2_FeSbO_6_	*P* = 3GPa	*P*2_1_/*n*	*T*_N_ = 60K	Possible *M* *E* coupling by theoretical calculation
	*T* = 1273K	(Mathieu *et al.*, 2013 ▶)		
CaMnTi_2_O_6_	*P* = 7GPa	*P*4_2_ *mc*	*T*_N_ = K	*A*-site ordering
	*T* = 1400K	(Aimi *et al.*, 2014 ▶)	*P* = 24Ccm^2^	
Pb_2_FeNdO_6_	*P* = 6GPa	*P*4_2_ *mc*	*T*_N_ = 150K	Evidence of *M* *E* coupling (Levstik *et al.*, 2008 ▶)
	*T* = 1500K	(Raevski *et al.*, 2013 ▶)		
CaMn_7_O_12_	*P* = 8GPa	*R*3	*T*_C_ = 90K	Can be synthesized @ AP
	*T* = 1273K	(Bochu *et al.*, 1974 ▶)	*P* = 2870Cm^2^ (Johnson *et al.*, 2012 ▶)	
BiMn_7_O_12_	*P* = 4GPa	*Im*	*T*_N_ = 22K	Evidence of *M* *E* coupling (Gauzzi *et al.*, 2013 ▶)
	*T* = 1273K	(Mezzadri *et al.*, 2009 ▶)	*T*_N_ = 55K	
PbMn_7_O_12_	*P* = 7.5GPa	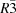	*T*_N_ = 68K	Evidence of *M* *E* coupling
	*T* = 1273K	(Locherer *et al.*, 2012 ▶)		
K_*x*_Fe_*x*_^2+^Fe^3+^_1*x*_F_3_	*P* = 1.3Kbar	*Pba*2	*T*_N_ = 118K	Complete MF
	*T* = 1053K	(Mezzadri *et al.*, 2008 ▶)		Hydrothermal synthesis
